# Evolutionary dynamics of the polyphenol oxidase gene family across plant lineages from algae to angiosperms

**DOI:** 10.1093/hr/uhag051

**Published:** 2026-02-18

**Authors:** Yanru Song, Kangkang Song, Boyuan Liu, Jingyu Wang, Haozhen Li, Qingqing Yan, Weiguang Zhang, Yiying Qi, Long Yang

**Affiliations:** College of Plant Protection and Agricultural Big-Data Research Center, Shandong Agricultural University, Taian 271018, China; College of Plant Protection and Agricultural Big-Data Research Center, Shandong Agricultural University, Taian 271018, China; College of Plant Protection and Agricultural Big-Data Research Center, Shandong Agricultural University, Taian 271018, China; College of Plant Protection and Agricultural Big-Data Research Center, Shandong Agricultural University, Taian 271018, China; College of Plant Protection and Agricultural Big-Data Research Center, Shandong Agricultural University, Taian 271018, China; College of Plant Protection and Agricultural Big-Data Research Center, Shandong Agricultural University, Taian 271018, China; College of Plant Protection and Agricultural Big-Data Research Center, Shandong Agricultural University, Taian 271018, China; College of Plant Protection and Agricultural Big-Data Research Center, Shandong Agricultural University, Taian 271018, China; College of Plant Protection and Agricultural Big-Data Research Center, Shandong Agricultural University, Taian 271018, China

Dear Editor,

Polyphenol oxidases (PPOs) are copper-binding enzymes that oxidize phenolics and play essential roles in plant development, secondary metabolism, and responses to biotic and abiotic stresses [1]. Structurally, PPO proteins typically harbor the conserved Tyrosinase, PPO1_DWL, and PPO1_KFDV domains, which are critical for enzymatic activity [2]. While PPO genes are common across plants, fungi, and insects, their distribution in plants is highly uneven, with substantial lineage-specific expansions and losses. Previous studies, mostly limited to dicots, have reported striking differences in PPO copy numbers—from abundant PPOs in bryophytes to complete absence in *Arabidopsis* [3]—yet the evolutionary origin, expansion mechanisms, and functional diversification of this family remain unclear. Here, we report a comprehensive evolutionary framework of PPO genes in 100 plant species representing major lineages from algae to angiosperms. In addition, we integrated these macroevolutionary findings with microevolutionary functional insights by profiling the expression of *Nicotiana tabacum* PPO genes under diverse abiotic stresses, thereby linking gene family evolution to potential regulatory specialization. This work advances our understanding of the evolution and diversification of PPO genes in plants and offers a foundation for functional studies and crop improvement.

Pfam, HMMER, and NCBI-CDD were used for genome-wide identification of PPO genes, resulting in the detection of 719 PPO genes across 82 species, whereas 18 species lacked detectable PPO copies. PPO copy numbers varied substantially among species, ranging from 0 to 71, with the highest number observed in *Alsophila spinulosa* (Fig. 1A). Notably, this study represents the first identification of PPO genes in algae, with two PPO genes detected exclusively in the *Chara braunii*. This finding challenges the previous hypothesis that PPO acquisition in land plants solely through horizontal gene transfer events from bacteria [4], and instead suggests an earlier origin dating back to the common ancestor of algae and land plants.

**Figure 1:**
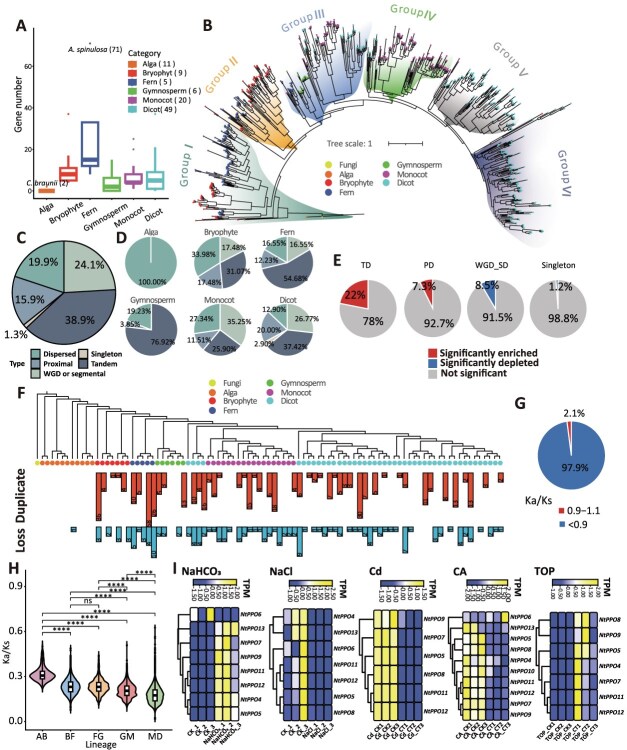
Evolutionary history and functional diversification of PPO genes. (A) Number of PPO genes identified in different plant lineages. Numbers in parentheses indicate the number of species in each lineage. *A. spinulosa* (71), PPO gene count in *Alsophila spinulosa* is 71; *C. braunii* (2), PPO gene count in *Chara braunii* is 2. PPO genes were identified using HMMERsearch with a stringent cutoff (*E*-value <1 × 10^−5^). (B) Phylogenetic tree of *PPO* genes constructed from 719 PPO proteins identified in 82 plant species, with a fungal PPO sequence used as an external outgroup. The phylogeny was inferred using the best-fit amino acid substitution model (WAG+R9), and node support was assessed with 1000 ultrafast bootstrap replicates. Based on overall tree topology and bootstrap support values greater than 50, PPO genes were classified into six major subgroups (Groups I–VI). Symbols at the tree tips represent different taxonomic lineages, and background shading denotes subgroup assignment. (C) Proportion of different duplication types of *PPO* genes across all species. (D) Proportion of *PPO* gene duplication types in alga, bryophyte, fern, gymnosperm, monocot, dicot. (E) Enrichment and depletion analysis of PPO duplication types relative to whole-genome backgrounds. TD, tandem duplication; PD, proximal duplication; WGD_SD, whole-genome and segmental duplication or segmental. Significance was assessed using Fisher’s exact test (*P* < 0.05). Enrichment and depletion were defined by enrichment ratios >1.2 and < 0.83, respectively. (F) Gene duplication and loss events were inferred using Notung v2.9 based on a TimeTree-derived species tree. Symbols represent species, red bars indicate duplications, and blue bars indicate losses. Numbers denote event counts. Species lacking reliable divergence-time information in TimeTree were excluded from this analysis to ensure topological consistency and analytical rigor. (G) Ka/Ks analysis of paralogous gene pairs. (H) Comparisons were conducted between successive plant lineages, including algae–bryophytes (AB), bryophytes–ferns (BF), ferns–gymnosperms (FG), gymnosperms–monocots (GM), and monocots–dicots (MD). Statistical significance was evaluated using the Wilcoxon rank-sum test. Asterisks denote significance levels: ns, not significant (*P* ≥ 0.05); *P* < 0.0001 (^****^). (I) Expression profiles of *Nicotiana tabacum* PPO genes (NtPPO) under abiotic stresses. Heatmap displays Log2 fold-change expression values under Cold (CA), Cadmium (Cd), Salt (NaCl), Alkaline (NaHCO₃), and Topping (TOP) treatments. RNA-seq datasets were obtained from the NCBI SRA database, with three biological replicates for both control and treatment groups. Gene expression levels were quantified as transcripts per million (TPM), and differential expression was determined using DESeq2 with thresholds of |log₂FoldChange| ≥ 0.5 and adjusted *P* ≤ 0.05. Reads were mapped to the reference genome with high efficiency, with mapping rates ranging from 97.06% to 99.02%.

To elucidate the evolutionary history and functional diversification of PPO genes across plant lineages, a maximum-likelihood phylogeny of PPO genes was reconstructed using IQ-TREE v2.4.0 based on 719 PPO proteins from 82 plant species, with a fungal PPO sequence included as an outgroup (Fig. 1B). Phylogenetic analysis classified PPOs into six subgroups, with early-diverging lineages enriched in Groups I–III and angiosperm-dominated Groups IV–VI retaining sporadic nonangiosperm members. This distribution pattern is consistent with, and suggests, vertical inheritance followed by lineage-specific expansion. Notably, the two PPO genes from the charophyte *C. braunii* clustered within Group I, immediately following the fungal outgroup and preceding bryophyte PPOs, a topology that further supports an early origin of PPO genes prior to the colonization of land.

Gene duplication analyses using MCScanX revealed lineage-specific mechanisms underlying PPO gene family expansion. Tandem duplication (TD) accounted for the largest proportion of PPO duplicates (38.9%), indicating that local gene amplification represents the dominant expansion mode (Fig. 1C). However, the relative contribution of duplication mechanisms varied markedly among plant lineages (Fig. 1D). Early-diverging lineages expanded PPOs mainly via dispersed duplication (DD), whereas TD played a greater role in ferns and gymnosperms. In angiosperms, whole-genome and segmental duplications (WGD) contributed to PPO expansion. Notably, TD-derived PPOs were significantly enriched relative to the genome background (Fig. 1E), suggesting sustained selective retention of locally duplicated genes during evolution.

Gene duplication types describe how PPO genes expanded, whereas duplication and loss analyses indicate gene retention and elimination during evolution. PPO gene duplication and loss events were inferred using Notung v2.9. Extensive duplication and loss events were detected across major plant lineages (Fig. 1F). In most lineages, duplication events exceeded gene losses, indicating an overall expansion tendency of the PPO gene family. Together, these results indicate that the present-day diversity and lineage-specific expansion of the PPO gene family are associated with heterogeneous duplication–loss dynamics, with recurrent TDs followed by differential retention contributing substantially to this process.

To investigate the selective pressures acting on PPO genes, Ka/Ks analyses were performed on both paralogous and orthologous gene pairs using ParaAT v2.0. Codon-based alignments were generated based on protein sequence alignments, and Ka, Ks, and Ka/Ks ratios were subsequently calculated. The majority of paralogous PPO pairs exhibited Ka/Ks ratios below 1, indicating that duplicated PPO genes have generally been subjected to purifying selection to maintain functional integrity (Fig. 1G). Similarly, Ka/Ks values for orthologous PPO gene pairs across plant lineages were consistently below 1, supporting strong functional conservation throughout plant evolution (Fig. 1H). Although Ka/Ks values remained consistently below 1, modest variation in their distributions was observed among lineages, suggesting differences in the strength of functional constraint following gene duplication and retention. Together, these results indicate that PPO gene family expansion was not accompanied by widespread divergence at the protein-coding level, and that duplicated PPO genes appear to have been largely preserved under stabilizing selection.

To link macroevolutionary patterns with functional specialization, we further examined the expression profiles of PPO genes in the representative dicotyledon *N. tabacum*. RNA-seq analyses under five treatments—cold, cadmium, salt, alkaline stress, and topping—revealed clear regulatory divergence among *NtPPO* genes (Fig. 1I). Clear regulatory divergence was observed among *NtPPO* genes. *NtPPO10* responded specifically to cold acclimation and was consistently downregulated, whereas *NtPPO6* showed pronounced sensitivity to ionic stresses, being strongly suppressed under salt and alkaline conditions. In contrast, *NtPPO11* and *NtPPO12* exhibited broad responsiveness across all treatments. Notably, this regulatory divergence occurs despite the strong purifying selection acting on PPO protein sequences, indicating that functional diversification within this family is more likely driven by changes in gene regulation rather than by extensive alterations of catalytic protein function. Based on their broad stress responsiveness, several NtPPO genes, including *NtPPO5*, *NtPPO8*, *NtPPO11*, and *NtPPO12*, represent promising candidates for future functional characterization. In this context, recent advances in genome editing technologies provide powerful tools to precisely manipulate gene regulation or expression levels, and targeted editing of key *NtPPO* genes may therefore offer a feasible strategy for enhancing stress tolerance in tobacco and related Solanaceae crops [5].

Taken together, our results establish an integrated evolutionary framework for the PPO gene family in plants. PPO genes originated prior to land colonization, as evidenced by their presence in algae, and subsequently underwent extensive lineage-specific expansion during plant evolution. This expansion was driven predominantly by TD, with additional contributions from dispersed and WGD in specific lineages. Despite repeated duplication events, most PPO genes were retained under strong purifying selection, preserving core enzymatic functions across plant lineages. In contrast, functional specialization of PPO genes appears to be driven predominantly by regulatory divergence rather than protein-coding sequence evolution, as reflected by stress-responsive expression divergence in *N. tabacum*. Together, these findings highlight a dual evolutionary strategy combining ancestral functional conservation with regulatory innovation, which has shaped the modern diversity and functional versatility of PPO genes and provides a foundation for future functional studies and targeted crop improvement.

## Abbreviations

DD, dispersed duplication; *NtPPO1, Nta03g28080; NtPPO2, Nta03g28100; NtPPO3, Nta04g22740; NtPPO4, Nta06g30520; NtPPO5, Nta15g17370; NtPPO6, Nta15g17450; NtPPO7, Nta16g13930; NtPPO8, Nta16g13960; NtPPO9, Nta16g14050; NtPPO10, Nta16g14130; NtPPO11, Nta19g09130; NtPPO12, Nta19g09140; NtPPO13, Nta24g03870;* PD, proximal duplication; PPO, polyphenol oxidase; TD, tandem duplication; WGD, whole-genome duplication

## Data Availability

The species information selected for this study is available at http://biodb.com.cn/PPO_Supplementary_Table/.
